# Associations between floods and bacillary dysentery cases in main urban areas of Chongqing, China, 2005–2016: a retrospective study

**DOI:** 10.1186/s12199-021-00971-z

**Published:** 2021-04-19

**Authors:** Yang Ma, Tong Wen, Dianguo Xing, Yan Zhang

**Affiliations:** 1 School of Public Health and Management, Research Center for Medicine and Social Development, Innovation Center for Social Risk Governance in Health, Chongqing Medical University, Yixueyuan Road, Yuzhong District, Chongqing, 400016 China; 2 Office of Health Emergency, Chongqing Municipal Health Commission, No.6, Qilong Road, Yubei District, Chongqing, 401147 China

**Keywords:** Floods, Bacillary dysentery, Distributed lag non-linear model, Relative risk, Attributable risk

## Abstract

**Background:**

Understanding the association between floods and bacillary dysentery (BD) incidence is necessary for us to assess the health risk of extreme weather events. This study aims at exploring the association between floods and daily bacillary dysentery cases in main urban areas of Chongqing between 2005 and 2016 as well as evaluating the attributable risk from floods.

**Methods:**

The association between floods and daily bacillary dysentery cases was evaluated by using distributed lag non-linear model, controlling for meteorological factors, long-term trend, seasonality, and day of week. The fraction and number of bacillary dysentery cases attributable to floods was calculated. Subgroup analyses were conducted to explore the association across age, gender, and occupation.

**Results:**

After controlling the impact of temperature, precipitation, relative humidity, long-term trend, and seasonality, a significant lag effect of floods on bacillary dysentery cases was found at 0-day, 3-day, and 4-day lag, and the cumulative relative risk (CRR) over a 7-lag day period was 1.393 (95%CI 1.216–1.596). Male had higher risk than female. People under 5 years old and people aged 15–64 years old had significantly higher risk. Students, workers, and children had significantly higher risk. During the study period, based on 7-lag days, the attributable fraction of bacillary dysentery cases due to floods was 1.10% and the attributable number was 497 persons.

**Conclusions:**

This study confirms that floods can increase the risk of bacillary dysentery incidence in main urban areas of Chongqing within an accurate time scale, the risk of bacillary dysentery caused by floods is still serious. The key population includes male, people under 5 years old, students, workers, and children. Considering the lag effect of floods on bacillary dysentery, the government and public health emergency departments should advance to the emergency health response in order to minimize the potential risk of floods on public.

**Supplementary Information:**

The online version contains supplementary material available at 10.1186/s12199-021-00971-z.

## Introduction

A series of extreme weather events caused by global climate change have posed a significant threat to public health. Extreme climate often leads to the prevalence of human diseases and results in the phenomenon that “a major epidemic occurs after a disaster” [1]. Floods are the most common and frequent extreme weather events. Besides, floods, as well as other social environmental factors, have an impact on public health involving fecal-oral transmitted infectious diseases, mesoporous infectious diseases, and insect-borne diseases [2, 3].

Bacillary dysentery is one of the intestinal infectious diseases caused by Shigella. According to the global disease burden report released by WHO in 2016, the disability-adjusted life expectancy of diarrheal diseases ranked fifth in the global disease burden [4]. According to a report of Chinese Center for Disease Control and Prevention, bacillary dysentery ranked third in the reported incidence of notifiable infectious diseases in China, and there were 250,000 to 500,000 reported cases per year between 2005 and 2010 [5].

A growing body of epidemiological evidences supported that floods will increase the risk of bacillary dysentery incidence [6, 7]. Case-crossover design was used to explore the relationship between floods and bacillary dysentery (OR 2.212 ~ 3.270) [8, 9], but the study period was relatively short, covering only one to three floods, and only OR values could be obtained. Liu et al. used distributed lag model to find out that the effect of floods on bacillary dysentery was statistically significant at lag 1 week (RR = 1.32, 95%CI 1.12–1.56), and the cumulated relative risk over 3-lag weeks period was 1.52 (95% CI 1.08–2.12) [10]. Milojevic et al. also used weekly morbidity data and got a negative conclusion after a strict control of confounding factors [11]. Ni et al. used monthly morbidity data and found the effect of floods on dysentery was shown with a 0-month lag [12]. The variability in the findings of these studies may be due to differences in climate and geographical environment at different study sites, or may be due to errors caused by insufficient accurate time scale of morbidity data. For infectious diseases like bacillary dysentery, with short incubation period and being susceptible to short-term acute effects of weather factors, Li et al. pointed out that when examining the effect of environmental factors, a more accurate time scale, such as daily data, should be considered in order to make the estimation more accurate [13–16]. In addition, when assessing population health risks due to flood, a high relative risk does not necessarily represent a high attributable disease burden due to impacts such as low exposure rates, whereas the attributable fraction (AF) can assess the disease burden due to risk factors more objectively by taking into account both the relative risk and exposure rates [17, 18].

Chongqing is located in the west of China, in the middle and upper Yangtze River, which is one of the highest risk levels in floods disasters [19]. Floods in Chongqing are characterized by rapid confluence, strong punching force, short duration, and frequent occurrence [20]. There has been no research on the association between floods and bacillary dysentery morbidity in Chongqing. Therefore, this study collected daily morbidity data of bacillary dysentery in the main urban area of Chongqing between 2005 and 2016 and aimed to evaluate the association between floods and daily bacillary dysentery cases in main urban areas of Chongqing over 7-lag day period with a distributed lag non-linear model. Besides, subgroup analysis was conducted to explore the susceptible population of bacillary dysentery caused by flood, and the attribution fraction and number were calculated to assess the disease burden.

## Materials and methods

### Study location

Chongqing is located in the west of China, in the middle and upper Yangtze River, which is one of municipalities under direct control of the central China’s government. And the terrain of Chongqing is mainly mountainous and hilly. The geographical scope of this study is the main urban area of Chongqing, which includes Yuzhong District, Jiangbei District, Shapingba District, Jiulongpo District, Nanan District, and Dadukou District, occupying 1.174 thousand square kilometers with a population of 5.0682 million by 2016, and the urbanization rate is 94.85%. The annual average temperature is between 16 °C and 18 °C, and the annual average precipitation ranges from 1000 to 1350 mm. Due to the impact of the subtropical monsoon climate, the floods could firstly happen in March, and end at November, mainly between June and September. Floods happened after September, which is extremely rare in the whole country [20].

### Data collection

#### Floods data and meteorological data

According to the Yearbooks of Meteorological Disasters in China, floods are defined as prolonged heavy rainfall or regional persistent rainfall (25.0–49.0 mm per day), rainstorm (daily precipitation ≥ 50.0 mm), and the local short time heavy rainfall, which cause river flooding and destroy buildings, thus causing geological disasters and results in economic losses and casualties. The floods events records were collected from the Yearbooks of Meteorological Disasters in China [21]. When the administrative regions of individual records were not available, we combined with the precipitation to assist judgment. Daily meteorological data were obtained from the National Meteorological Science Data Center [22], which included daily mean temperature, daily relative humidity, and daily mean precipitation.

#### Bacillary dysentery data and demographic data

Bacillary dysentery is caused by Shigella, one of intestinal infectious diseases with main clinical manifestations like fever, diarrhea, and abdominal pain. Bacillary dysentery is infected mainly through fecal-oral transmission, and it’s the legal B class infectious disease in China. We obtained and verified daily disease surveillance data on bacillary dysentery between 2005 and 2016 from the National Notifiable Disease Surveillance System (NNDSS). In our study, all bacillary dysentery cases were defined based on the diagnostic criteria and principles of management for dysentery (WS 287-2008) launched by the National Health Commission of the People’s Republic of China [23]. Only the cases confirmed clinically and by laboratory tests were included in this research. According to the National Communicable Disease Control Act, doctors must report each bacillary dysentery case to the local health departments, and the local health departments should report these cases to the highest level within 24 h to ensure the consistency of all cases of monitoring over the study period [24]. Demographic data of Chongqing were obtained from Chongqing Statistical Yearbook [25].

### Statistical analysis

#### Descriptive analysis

In this study, the annual bacillary dysentery morbidity between 2005 and 2016 was calculated, and its change was tracked during 12 years. Time sequence plots were used to depict the distribution of daily bacillary dysentery cases and the temporal change of floods. The descriptive analyses of daily bacillary dysentery cases and meteorological data were conducted by calculating the maximum, minimum, quartile, median, and mean value.

#### Regression analysis

Distributed lag non-linear regression model (DLNM) was applied to investigate the association between floods and bacillary dysentery. Considering the effects of temperature, relative humidity, and precipitation to the incidence of bacillary dysentery [17, 26, 27], both immediate and lag effect of temperature on bacillary dysentery incidence were controlled in the model [10]; relative humidity and precipitation were controlled through natural cubic spline function. Firstly, a regression model was constructed with all data from 2005 to 2016. Secondly, the study period is divided into two periods, 2005–2010 and 2011–2016, and according to the different trends of annual incidence rate, two regression models were constructed to explore the effect of floods on the bacillary dysentery over time. Finally, subgroup analyses were conducted to investigate the risk of bacillary dysentery among different populations (gender, age, and occupation). Based on a generalized linear model (GLM), the regression model is as follows:

Model:

$$ E\left[g\left({Y}_t\right)\right]=\alpha +\sum \limits_{l={l}_0}^L{\beta}_l{\mathrm{flood}}_{t-l}+\sum \limits_{l={l}_0}^L{\gamma}_l{AT}_{t-l}+ ns\left({\mathrm{precipitation}}_t\right)+ ns\left({ARH}_t\right)+ ns\left(\mathrm{time}\right)+{\mathrm{dow}}_t $$

In the model, *Y*_*t*_ represents daily bacillary dysentery cases, *t* represents time, *l* represents lag days. The maximum lag day is set at 7. “flood” is coded as categorical variable, with 1 refers to flood and 0 refers to non-flood. AT represents daily mean temperature, precipitation represents daily mean precipitation, ARH represents daily mean relative humidity, ns represents natural cubic spline, and dow is an indicator variable representing the day of week. The collinearity between lag days is reduced by fitting polynomial function [28], and natural cubic spline is used to control the non-linear effect of precipitation and relative humidity on bacillary dysentery incidence (both the degrees of freedom are set at 3). Besides, a natural cubic spline with 7 df/year is set to control long-term trend and seasonality of time [17, 26, 29].

#### Attributable risk with the framework of DLNM

The attributable fraction (AF) is the proportion of cases that would not have occurred in the absence of exposure among the exposed population. The attributable number (AN) can be calculated by the total exposed population and AF. Traditional estimation of AF ignores the complexity of non-linear correlation and potential lag effects in time series analysis. In the distributed lag non-linear model, a lag dimension is added to the exposure-response relationship through the cross-basis function, thus obtaining the double dimension exposure-lag-response relationship function: ${\beta}_{x_{t-l},l}$, which can simultaneously assess the delayed effect and non-linear effect of exposure factors [30]. The calculation formula of attribution risk based on DLNM is as follows:

$$ {\mathrm{AF}}_{x,t}=1-\exp \left(-{\sum}_{l={l}_0}^L{\beta}_{x_{t-l},l}\right) $$

There are two methods to estimate AF based on DLNM: forward and backward perspective. “Forward perspective” indicates risk in a future period time (*t* + *l*_0_,…,*t* + *L*) due to current exposure at time *t* [31], and “backward perspective” estimates the risk at time *t* attributed to a series of exposure events in the past (*t* − *l*_0_,…,*t* − *L*) [32, 33]. We reported the AF and AN of daily bacillary dysentery cases to floods from both forward and backward perspective.

All statistical analyses were performed using R version 3.3.2 (The R Project for Statistical Computing, Vienna, Austria).

## Results

### Descriptive analysis

**Fig. 1:**
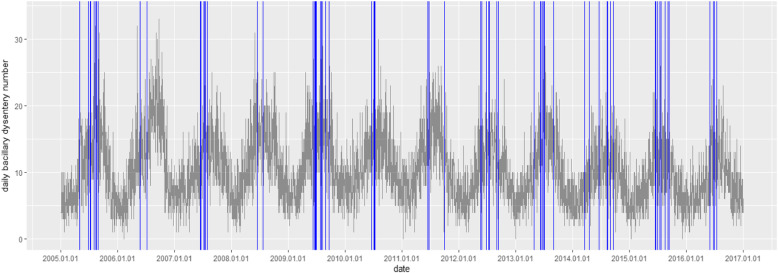
Distribution of daily bacillary dysentery cases during study period from 2005 to 2016 in main urban areas of Chongqing, China (Day with flood is indicated as blue line.)

Between 2005 and 2016, there were 45,691 diagnosed bacillary dysentery cases, including 23,757 males and 21,934 females. The annual incidence of bacillary dysentery was 84.03/10^5^. During 2005–2010 and 2011–2016, the annual incidence showed distinctly different characteristics: between 2005 and 2010, the annual incidence fluctuated between higher levels of 90.80/10^5^ and 104.56/10^5^; while from 2011 until 2016, the annual incidence decreased steadily from 87.93/10^5^ to 63.14/10^5^ (Supplementary Table 1). The median daily bacillary dysentery case was 10 and the maximum was 34. During the study period, a total of 56 floods occurred in the urban area of Chongqing. The daily mean precipitation was 3.13 mm and the maximum was 271 mm. The daily mean temperature was 19.01 °C and the daily mean relative humidity was 0.76. (Table 1 and Fig. 1)

**Table 1:** Descriptive analysis of variables

Variables	Min	P25	Median	Mean	P75	Max
**Precipitation**	0.00	0.00	0.00	3.13	1.40	271.00
**AT**	1.20	11.95	19.30	19.01	25.20	36.70
**ARH**	0.29	0.69	0.78	0.76	0.86	1.00
**Bacillary dysentery case**						
**Total**	0	7	10	10.42	14	34
**Gender**						
Male	0	3	5	5.42	7	21
Female	0	3	5	5.00	7	18
**Age**						
0 ~ 4	0	2	4	4.38	6	28
5 ~ 14	0	0	0	0.68	1	7
15 ~ 64	0	2	4	4.41	6	27
65~	0	0	1	0.95	1	9
**Occupation**						
Student	0	0	1	1.00	2	9
Farmer	0	0	0	0.44	1	7
Worker	0	0	1	0.87	1	8
Child	0	2	4	4.59	6	28
Other	0	2	3	3.52	5	22

Note: *Min*, minimum; *P25*, the 25th percentile; *P75*, the 75th percentile; *Max*, maximum; *AT*, daily mean temperature; *ARH*, daily mean relative humidity

### Regression analysis

**Fig. 2:**
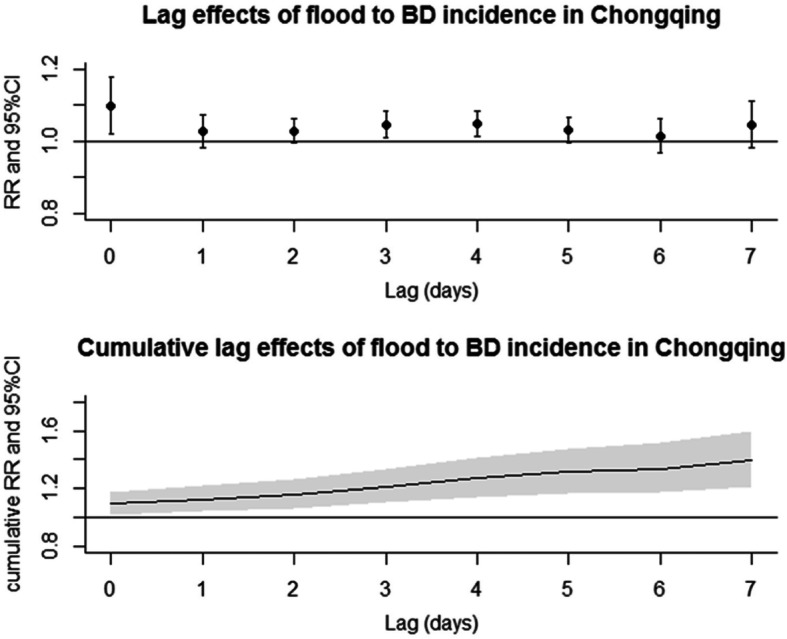
Distributed lag non-linear effects of floods to bacillary dysentery incidence in Chongqing

After controlling temperature, relative humidity, precipitation, long-term trend, and seasonality of time, the effect of floods on the incidence of bacillary dysentery was statistically significant at lag 0-day, 3-day, and 4-day lag, and the cumulative relative risk (CRR) over a 7-day period was 1.393 (95% CI 1.216–1.596). The CRR for female was 1.272 (95% CI 1.057–1.531) and 1.515 (95% CI 1.262–1.818) for male. The CRR for children under 5 years old and people aged in 15 ~ 64 years old were 1.266 (95% CI 1.040–1.541) and 1.534 (95%CI 1.252–1.878). The CRR for students, workers, and children were all statistically significant (Table 2 and Fig. 2)

**Table 2:** Cumulative relative risk based on 7-lag distributed model

Group	CRR	95%CI
**Total**	**1.393**	**1.216–1.596**
**Gender**		
**Female**	**1.272**	**1.057–1.531**
**Male**	**1.515**	**1.262–1.818**
**Age**		
**0 ~ 4**	**1.266**	**1.040–1.541**
5 ~ 14	1.398	0.818**–**2.392
**15 ~ 64**	**1.534**	**1.252–1.878**
65~	1.371	0.873**–**2.151
**Occupation**		
**Student**	**1.857**	**1.196–2.884**
Farmer	1.066	0.595**–**1.909
**Worker**	**1.593**	**1.040–2.441**
**Child**	**1.300**	**1.071–1.579**
**Other**	**1.419**	**1.132–1.779**

Note: *CRR*, cumulative relative risk; *CI*, confidence interval; *P* value < 0.05 for bold figures

In Table 3, compared to the 2005–2010 period, the CRR of floods to bacillary dysentery incidence for the whole population, male and people aged between 15 and 64 years old increased in the 2011–2016 period. The CRR for children under 5 years old increased from 1.197 (95%CI 0.882–1.624) to 1.293 (95%CI 0.995–1.680), while it decreased from 1.394 (95%CI 1.091–1.780) to 1.104 (95%CI 0.828–1.472) for female (Table 3).

**Table 3:** The cumulative relative risk of floods to bacillary dysentery incidence during 2005–2010 and 2011–2016

Year period	2005–2010		2011–2016	
**Group**	**CRR**	**95%CI**	**CRR**	**95%CI**
**Total**	**1.371**	**1.143–1.645**	**1.398**	**1.136–1.720**
**Gender**				
**Female**	**1.394**	**1.091–1.780**	1.104	0.828**–**1.472
**Male**	**1.350**	**1.058–1.723**	**1.714**	**1.302–2.257**
**Age**				
0 ~ 4	1.197	0.882**–**1.624	1.293	0.995**–**1.680
5 ~ 14	1.464	0.770**–**2.789	0.992	0.356**–**2.764
**15 ~ 64**	**1.427**	**1.114–1.829**	**1.731**	**1.207–2.483**
65~	1.575	0.890**–**2.786	1.103	0.522**–**2.332
**Occupation**				
Student	1.613	0.932**–**2.792	2.075	0.963**–**4.472
Farmer	1.279	0.640**–**2.556	0.512	0.158**–**1.660
**Worker**	**1.737**	**1.054–2.862**	1.209	0.510**–**2.865
Child	1.323	0.982**–**1.781	1.266	0.976**–**1.642
Other	1.262	0.955**–**1.669	**1.759**	**1.192–2.595**

Note: *CRR*, cumulative relative risk; *CI*, confidence interval; *P* value < 0.05 for bold figures

### Bacillary dysentery incidence risk attributable to floods

The estimated AF and AN value of the two methods were similar (Table 4), and the results of the backward method will be described and discussed in this paper as an example. In the study area, during the period between 2005 and 2016, the attributable fraction of bacillary dysentery incidence to floods was 1.10% over 7-day lag periods, and attributable number was 497 people. The AF and AN of males were higher than that of females, and the AF and AN of people under 5 years old and people aged between 15 and 64 years old were statistically significant. Compared with 2005–2010 period, the attributable risk of bacillary dysentery incidence to floods was increased for male and people under 5 years old in 2011–2016 period. The AF and AN for male were increased by 0.76% and 67 persons separately; and the AF and AN for people under 5 years old was increased by 0.13% and 56 persons separately (Supplementary Table 2).

**Table 4:** Attributable fraction and number of bacillary dysentery incidence to floods between 2005 and 2016

Group	Forward perspective		Backward perspective	
	AF (%)	AN (n)	AF (%)	AN (n)
**Total**	0.98(0.63–1.27)	440(279–578)	1.10(0.62–1.50)	497(294–605)
**Gender**				
Male	1.18(0.75–1.59)	281(171–373)	1.36(0.80–1.92)	324(187–453)
Female	0.73(0.22–1.20)	159(37–256)	0.79(0.22–1.35)	173(40–296)
**Age**				
0 ~ 4	1.03(0.14–1.30)	197(18–245)	1.15(0.15–1.46)	220(34–284)
5 ~ 14	0.83(− 0.71–1.71)	24(− 18–51)	0.94(− 0.60–2.20)	28(− 17–63)
15 ~ 64	0.98(0.68–1.61)	188(134–312)	1.10(0.75–2.03)	211(140–388)
65~	0.79(− 0.46–1.5)	33(− 17–61)	0.89(− 0.45–1.83)	37(− 16–79)
**Occupation**				
Student	0.84(0.50–1.92)	37(25–85)	0.96(0.53–2.63)	42(19–120)
Farmer	1.00(− 2.34–1.74)	19(− 50–31)	1.15(− 1.92–2.19)	22(− 38–44)
Worker	0.99(0.09–2.08)	38(5–79)	1.12(0.16–2.69)	43(5–106)
Child	1.02(0.18–1.28)	206(50–266)	1.14(0.22–1.56)	229(41–313)
Other	0.93(0.36–1.43)	144(60–227)	1.04(0.43–1.73)	161(62–274)

Note: *AF*, attributable fraction; *AN*, attributable number

## Discussion

This study found the association between floods and daily bacillary dysentery cases in main urban area of Chongqing, China. The cumulative risk of floods on bacillary dysentery incidence was 1.393 based on a 7-day distributed lag non-linear model. The result was basically consistent with relative risk from studies that evaluated association between extreme weather events and infectious diseases by using daily cases [34, 35], while lower than relative risk based on weekly or monthly cases [13, 36]. Considering the short incubation period and acute onset of bacillary dysentery, daily case instead of weekly or monthly case was used to quantify the correlation between floods and incidence of bacillary dysentery, which could result in higher precision and accuracy of evaluation [13–16].

We found that floods played an impact on bacillary dysentery incidence in 0-day, 3-day, and 4-day lag. Factors affecting the length of lag effect include suitable microenvironment for pathogen growth, transmission pathways (mainly for people exposed to contaminated water or food), and basic sanitation facilities [37]. Floods in Chongqing are characterized by rapid confluence, strong punching force, short duration, and frequent occurrence [20]. As a result, the wet microenvironment suitable for the growth of Shigella exists short, and the duration of population exposure to floods is short. Coupled with the short incubation period of bacillary dysentery, which is only 1–3 days generally, it is likely that the exposed population will develop bacillary dysentery 3 and 4 days after the flood, showing a significant effect of a 3-day and 4-day lag. The people with a 0-day lag in onset of disease may be those with poorer immunity. When they are exposed to the germs and then affected by the emergency floods, resulting in acute psychological stress, leading to a further decrease in immunity [38], thus the onset of disease may be more acute. The short exposure time and short lag effect made bacillary dysentery cases attributed to floods few, with an attributable fraction of 1.10% and attributable number of 497. However, the higher relative risk indicates that the risk of floods to bacillary dysentery incidence is still high. The impact of floods on bacillary dysentery incidence in population is severe in a short time, suggesting that the government and health emergency departments should be well prepared in the early warning of the onset of bacillary dysentery during floods.

The floods affect people’s health through influencing the transmission and spread of pathogen. The humid environment during the floods is conducive to the breeding and growth of Shigella and increases Shigella contamination opportunities for vegetable and fruits through water transmission [36]. In Chongqing, people like eating raw or cold food in summer and have more chance to eat out or eat takeout, so they may have contact with Shigella-contaminated food more easily and thus suffer from bacillary dysentery during floods [39]. Further subgroup analysis revealed a higher risk of bacillary dysentery caused by floods in males, students, and workers. And the CRR value in male was 1.515, which was higher than that in female. Compared with female, the higher susceptibility of male may be due to the greater range of outings, more opportunities for outdoor work, and more involvement in flood relief operations, which increases male’s infection chance with pathogens during floods and increases risk of suffering from bacillary dysentery [9]. For students, concentrated accommodation and diet, coupled with poor hygiene awareness, outside diet or more snacks make them more easily infected by Shigella during floods [40]. In 2012, there were 12 outbreaks of bacillary dysentery reported in mainland China and 11 occurred in schools, of which 5 were foodborne and 4 were waterborne. The reasons were related to the contamination of school drinking water or food by Shigella. Therefore, the occurrence of floods may increase the probability of contamination and students’ risk of suffering from bacillary dysentery. Therefore, it is necessary to strengthen the health supervision of schools, school surrounding, and meal delivery agencies during floods in order to reduce the risk of bacillary dysentery among students [40, 41]. Workers are one of the vulnerable groups affected by floods, with a cumulative relative risk of 1.593. This may be due to poor accommodation conditions, poor sanitary conditions, and weak flood control capacity, combined with the poor living habits of workers themselves, thus increasing workers’ exposure to contaminated water or food during floods and develop bacillary dysentery [42]. In addition, compared to the above population, children under 5 years old, although with a lower cumulative relative risk (CRR = 1.266, 95% CI 1.041–1.541), had a higher number (220) of bacillary dysentery attributed to floods, accounting for almost half of the total AN, which is due to the high total number of bacillary dysentery cases in this population.

In this study, we found that the annual incidence rate of bacillary dysentery in the main urban area of Chongqing fluctuated at high levels from 2005 to 2010, while after 2011, the annual incidence rate decreased year by year, indicating that the overall risk of bacillary dysentery in the population decreased. This may be related to the improved sanitary conditions and human interventions, such as timely diagnosis and treatment of bacillary dysentery patients, brought about by the rapid socioeconomic development in the last decade [43, 44]. However, compared to 2005–2010, the CRR and AF of bacillary dysentery due to floods increased slightly from 2011 to 2016, especially for the male population, where the CRR and AF of bacillary dysentery due to floods increased obviously, from 1.350 and 1.01% to 1.714 and 1.77%, respectively. Thus, although the annual incidence rate decreased in the latter period, the AN in male increased significantly, from 129 to 196, indicating that not only the relative risk but also the absolute risk is increasing in this population. In this regard, on the one hand, it may be due to the still weak flood resilience in urban areas. As a result of rapid urbanization, the expansion of urban buildings and non-seepage surfaces reduces the infiltration of rainwater into the soil, increases the volume and rate of surface runoff, and creates a burden on the drainage system [45, 46], further increasing flood frequency in urban areas and making these areas more vulnerable. On the other hand, with the increasing frequency of population movements, the urban population is becoming denser and the number of people exposed to floods increases, thus the opportunities for the spread and infection of Shigella increase [47]. Studies have shown that the population vulnerability of China’s floods has gradually increased, with Chongqing growing at the fastest rate [19]. In contrast to male, the CRR, AF, and AN in female decreased obviously (from 1.394, 1.14%, and 133 to 1.104, 0.31%, and 32, respectively), which may be related to the change of family labor structure in China. With the improvement of the level of social and economic productivity in China, the family’s livelihood can be sustained by the couple’s unilateral supply [48]. Under the influence of traditional gender culture “men take care of outside the house while women take care of inside the house” and the introduction of universal two-child policy in China, women reduce working hours or even give up employment to take care of their children. The labor participation of women has declined [49, 50], thus reducing the chance of going out to contact pathogen during floods. To sum up, with the change and evolution of urban humanistic factors, the preventive measures against bacillary dysentary caused by floods should be improved and strengthened accordingly, such as improving the prevention measures of key population, improving comprehensive flood resistance ability of cities, and ensuring the sanitary and drinking water hygiene of residents during floods.

The first limitation of this study is that the effect of social and economic conditions, medical and health facilities, access to health services, and other factors on the incidence of bacillary dysentery are not quantified. The second is that this study used data from the infectious disease network direct reporting system, and some people with bacillary dysentery but self-healing were not included in the scope of this study. As a result, the incidence of bacillary dysentery in the population may be underestimated.

## Conclusions

This study confirmed that floods would increase the risk of bacterial dysentery in the population within the fine time scale, and the risk of bacillary dysentery in the main urban area of Chongqing caused by floods is still serious. The key population includes male, people under 5 years old, students, workers, and children. Considering the lag effect of floods on bacillary dysentery, the government and public health emergency departments should advance the emergency health response in order to minimize the potential risk of floods to the public.

## Supplementary Information

**Additional file 1: Supplementary Table 1** Annual incidence of bacillary dysentery between 2005-2016. **Supplementary Table 2** Attributable fraction and number of bacillary dysentery incidence to floods between 2005-2010, 2011-2016.

## Data Availability

The data of bacillary dysentery cases analyzed during the study are not publicly available due to regulations, but they can be obtained from the corresponding author on reasonable request.

## Abbreviations

AF: Attributable fraction
AN: Attributable number
BD: Bacillary dysentery
CI: Confidence interval
CRR: Cumulative relative risk
DLNM: Distributed lag non-linear regression model
GLM: Generalized linear model
NNDSS: National Notifiable Diseases Surveillance System
OR: Odds ratio
RR: Relative risk
WHO: World Health Organization
