# Supplementary material for: Associations between floods and bacillary dysentery cases in main urban areas of Chongqing, China, 2005–2016: a retrospective study
Source: Environ Health Prev Med. 2021 Apr 19;26:49. doi: 10.1186/s12199-021-00971-z (PMC8056597; doi:10.1186/s12199-021-00971-z)
Supplement: Supplementary file 1 — Additional file 1: Supplementary Table 1 Annual incidence of bacillary dysentery between 2005-2016. Supplementary Table 2 Attributable fraction and number of bacillary dysentery incidence to floods between 2005-2010, 2011-2016. [file 12199_2021_971_MOESM1_ESM.docx]

**Supplementary Table 1 Annual incidence of bacillary dysentery during 2005-2016.**

| **Year** | **2005** | **2006** | **2007** | **2008** | **2009** | **2010** | **2011** | **2012** | **2013** | **2014** | **2015** | **2016** |
| --- | --- | --- | --- | --- | --- | --- | --- | --- | --- | --- | --- | --- |
| **Annual incidence（1/10*^5^*）** | 92.58 | 104.56 | 90.80 | 95.37 | 98.05 | 91.44 | 87.93 | 75.07 | 76.40 | 69.41 | 63.59 | 63.14 |

**Supplementary Table 2 Attributable fraction and number of bacillary dysentery incidence to floods during 2005-2010, 2011-2016**

| **Group** | **Forward perspective** | |  | **Backward perspective** | |
| --- | --- | --- | --- | --- | --- |
|  | **AF (%)** | **AN(n)** |  | **AF (%)** | **AN(n)** |
| **2005-2010** |  |  |  |  |  |
| **Total** | 0.95(0.63-1.27) | 233(279-578) |  | 1.07(0.48-1.63) | 261(110-410) |
| **Gender** |  |  |  |  |  |
| Female | 1.02(0.36-1.58) | 119(37-178) |  | 1.14(0.28-1.86) | 133(32-220) |
| Male | 0.90(0.21-1.44) | 114(23-186) |  | 1.01(0.25-1.74) | 129(24-223) |
| **Age** |  |  |  |  |  |
| 0~4 | 0.96(-0.44-1.36) | 74(-28-106) |  | 1.05(-0.45-1.58) | 81(-32-122) |
| 5~14 | 0.92(-1.00-2.20) | 17(-20-40) |  | 1.07(-5.98-2.39) | 20(-15-54) |
| 15~64 | 0.99(0.42-1.69) | 123(48-208) |  | 1.12(0.75-2.03) | 139(61-257) |
| 65~ | 0.81(-0.35-1.86) | 20(-9-47) |  | 0.89(-0.39-2.51) | 22(-9-64) |
| **Occupation** |  |  |  |  |  |
| Student | 0.84(-0.09-1.99) | 23(-5-55) |  | 1.01(-0.23-2.86) | 28(-3-77) |
| Farmer | 1.08(-2.34-2.40) | 14(-28-32) |  | 1.26(-1.97-3.38) | 16(-26-43) |
| Worker | 1.01(0.18-2.46) | 27(10-65) |  | 1.16(0.31-3.44) | 31(7-91) |
| Child | 0.97(-0.34-1.48) | 79(-6-127) |  | 1.07(-0.04-1.62) | 87(-9-131) |
| Other | 0.94(-0.12-1.41) | 89(-10-130) |  | 1.04(-0.16-1.63) | 98(-20-161) |
| **2011-2016** |  |  |  |  |  |
| **Total** | 0.95(0.39-1.40) | 203(82-295) |  | 1.08(0.44-1.66) | 229(90-362) |
| **Gender** |  |  |  |  |  |
| Female | 0.30(-0.58-1.01) | 31(-69-104) |  | 0.31(-0.57-1.14) | 32(-73-117) |
| Male | 1.46(0.81-1.97) | 162(92-220) |  | 1.77(0.85-2.56) | 196(101-280) |
| **Age** |  |  |  |  |  |
| 0~4 | 1.06(-0.02-1.52) | 122(-10-170) |  | 1.18(-0.05-1.79) | 137(-6-204) |
| 5~14 | 0.63(-3.74-1.37) | 7(-51-16) |  | 0.72(-2.80-1.89) | 8(-34-22) |
| 15~64 | 0.89(0.56-1.86) | 61(43-130) |  | 1.01(0.51-2.49) | 69(40-177) |
| 65~ | 0.74(-2.28-1.41) | 13(-39-26) |  | 0.85(-1.89-1.97) | 15(-35-33) |
| **Occupation** |  |  |  |  |  |
| Student | 0.78(-0.17-2.16) | 13(-3-34) |  | 0.92(-0.11-3.43) | 15(-3-54) |
| Farmer | 0.77(-14.38-1.02) | 5(-92-8) |  | 0.88(-6.29-1.56) | 6(-42-10) |
| Worker | 0.85(-3.20-1.98) | 10(-34-22) |  | 0.97(-2.48-2.76) | 11(-26-29) |
| Child | 1.04(-0.11-1.43) | 124(-10-172) |  | 1.17(-0.12-1.66) | 139(-9-199) |
| Other | 0.87(0.49-1.87) | 52(30-112) |  | 0.98(0.54-2.56) | 59(32-150) |

Note: AF, attributable fraction; AN, attributable number.
